# Identification of hypoxanthine as a urine marker for non-Hodgkin lymphoma by low-mass-ion profiling

**DOI:** 10.1186/1471-2407-10-55

**Published:** 2010-02-23

**Authors:** Byong Chul Yoo, Sun-Young Kong, Sang-Geun Jang, Kyung-Hee Kim, Sun-A Ahn, Weon-Seo Park, Sohee Park, Tak Yun, Hyeon-Seok Eom

**Affiliations:** 1 Colorectal Cancer Branch, Division of Translational and Clinical Research I, Research Institute, National Cancer Center, Goyang-si, Republic of Korea; 2 Hematologic Malignancies Branch, Division of Translational and Clinical Research II, Goyang-si, Republic of Korea; 3 Cancer Biostatistics Branch, Division of Epidemiology & Management, Research Institute, National Cancer Center, Goyang-si, Republic of Korea; 4 Hematology-Oncology Clinic, Center for Specific Organs Cancer, National Cancer Center, Goyang-si, Republic of Korea

## Abstract

**Background:**

Non-Hodgkin lymphoma (NHL) is a hematologic malignancy for which good diagnostic markers are lacking. Despite continued improvement in our understanding of NHL, efforts to identify diagnostic markers have yielded dismal results. Here, we translated low-mass-ion information in urine samples from patients with NHL into a diagnostic marker.

**Methods:**

To minimize experimental error, we tested variable parameters before MALDI-TOF analysis of low-mass ions in urine. Urine from 30 controls and 30 NHL patients was analyzed as a training set for NHL prediction. All individual peak areas were normalized to total area up to 1000 m/z. The training set analysis was repeated four times. Low-mass peaks that were not affected by changes in experimental conditions were collected using MarkerView™ software. Human Metabolome Database (HMDB) searches and ESI LC-MS/MS analyses were used to identify low-mass ions that exhibited differential patterns in control and NHL urines. Identified low-mass ions were validated in a blinded fashion in 95 controls and 66 NHL urines to determine their ability to discriminate NHL patients from controls.

**Results:**

The 30 highest-ranking low-mass-ion peaks were selected from the 60-urine training set, and three low-mass-ion peaks with high intensity were selected for identification. Of these, a 137.08-m/z ion showed lower mass-peak intensity in urines of NHL patients, a result that was validated in a 161-urine blind validation set (95 controls and 66 NHL urines). The 130.08-m/z ion was identified from HMDB searches and ESI LC-MS/MS analyses as hypoxanthine (HX). The HX concentration in urines of NHL patients was significantly decreased (P < 0.001) and was correlated with the mass-peak area of the 137.08-m/z ion. At an HX concentration cutoff of 17.4 μM, sensitivity and specificity were 79.2% and 78.4%, respectively.

**Conclusions:**

The present study represents a good example of low-mass-ion profiling in the setting of disease screening using urine. This technique can be a powerful non-invasive diagnostic tool with high sensitivity and specificity for NHL screening. Furthermore, HX identified in the study may be a useful single urine marker for NHL screening.

## Backgrounds

Non-Hodgkin lymphomas (NHL) are a heterogeneous group of malignancies that arise from lymphoid tissue. They exhibit varied clinical and biological features [1], and their incidence has been increasing over the past several decades [2]. The past decade has seen enormous changes in our understanding of lymphomas, including the identification of better prognostic factors [3]. However, results from efforts to identify good *diagnostic *factors have been disappointing. Lactate dehydrogenase has been used as an NHL marker [4], but its accuracy in diagnosis has been unsatisfactory. Thus, finding specific tumor markers that are useful for diagnosing and monitoring NHL remains a high priority. In the present study, we introduce a new NHL diagnostic marker obtained by translating the information in low-mass ions (i.e., < 1000 m/z) in urine samples from lymphoma patients.

## Methods

### Urine from patients with NHL

Urine samples were obtained from 125 healthy controls (65 males and 60 females, median age 50.0 years old) and 96 patients with NHL (61 males and 35 females, median age 57.0 years old). All samples were stored at 4°C before processing and were processed within 6 hours of collection. Processed samples were frozen at -80°C until assessment. The characteristics of NHL patients are listed in Table 1. Informed consent was obtained from all patients, and the research protocol was approved by the Institutional Review Board of the National Cancer Center, Korea (NCCCTS-05-146).

**Table 1:** The characteristics of 96 NHL patients

	No. of Patients (%)
Age, years (median, range)	57, 23-87
Sex	
Male	61 (63.5)
Female	35 (36.5)
Histologic type (WHO)	
Diffuse large B cell lymphoma	70 (72.9)
Mantle cell lymphoma	6 (6.2)
T cell lineage	16 (16.7)
Follicular lymphoma	2 (2.1)
Burkitt's lymphoma	2 (2.1)
Performance status	
0	32 (33.3)
1	60 (62.5)
2	4 (4.2)
Stage	
I	16 (16.7)
II	40 (41.6)
III	18 (18.8)
IV	22 (22.9)
Extra-nodal involvement	
Absent	43 (44.8)
Present	57 (55.2)
IPI	
Low	52 (54.1)
Low-intermediate	18 (18.8)
High-intermediate	20 (20.8)
High	6 (6.3)
Bone marrow involvement	
Absent	90 (93.8)
Present	5 (5.2)
Not identified	1 (1.0)
Therapeutic regimen	
R-CHOP	79 (82.3)
CHOP	8 (8.3)
Others	9 (9.4)

In histologic type, T cell lineage includes anaplastic large cell lymphoma, angioimmunoblastic T cell lymphoma, peripheral T cell lymphoma, and NK/T cell lymphoma. Except of follicular lymphoma as an indolent type of NHL, all other histologic subclasses represent aggressive type.

Performance status was determined according to the criteria, ECOG score.

IPI: international prognostic index

CHOP regimen includes cyclophosphamide, doxorubicin, vincristine, and prednisolone. R-CHOP is a combination regimen of CHOP with the anti-CD20 monoclonal antibody, rituximab.

### MALDI-TOF analytical conditions for collecting low-mass ions in urine

Urine samples were mixed (1:12) with an α-cyano-4-hydroxycinnamic acid solution in 50% acetonitrile/0.1% trifluoroacetic acid (TFA). Differences between normal and cancer urine samples were determined using a 4700 Proteomic Analyzer (Applied Biosystems, Foster City, CA, USA). The mass-spectral data represent the average of 20 accumulated spectra.

### Low-mass ion selection and statistical analysis

All MALDI mass spectra, formatted as *.t2d files, were analyzed with MarkerView™ Software version 1.2 (Applied Biosystems/MDS Sciex, Toronto, Canada). The optimized parameters used to compare low-mass peaks in urines from controls and NHL patients were as follow: Mass tolerance, 100 ppm; minimum required response, 100; maximum number of peaks, 5000; normalization, by total area sums. After collecting information from MALDI mass spectra, principal component analyses (PCA) and t-tests were used to select low-mass ions with differential peak intensities in urines from controls and NHL patients.

### ESI-MS/MS for low-mass ion analysis

MALDI-TOF was not suitable for comparing MS/MS patterns of low-mass ions, so ESI-MS/MS was employed. The mass spectrometer was set for ESI in positive mode. A syringe pump was used to introduce the calibration solution for automatic tuning and calibration of the LTQ-XL (Thermo Fisher Scientific Inc., Waltham, MA) in ESI positive-ion mode. Standard solutions (1 μM hypoxanthine) were infused directly into the ionization source of the mass spectrometer using a syringe pump (1.0 μL/min) without chromatographic separation. The spray voltage was set at +1.1 kV; the temperature of the capillary was set at 200°C; the capillary voltage was set at +20 V; the tube lens voltage was set at +100 V; and the auxiliary gas was set to zero. Full-scan experiments were performed to linear trap in the range, 100-200 m/z. Systematic MS/MS experiments were performed by changing the relative collisional energy and monitoring the intensities of the fragment ions. MS/MS data were acquired from urine samples.

### Dtermination of hypoxanthine and xanthine in urines

The concentration of hypoxanthine and xanthine in urines was determined using the Amplex^® ^Red Xanthine/Xanthine Oxidase Assay Kit (Molecular Probes, Inc., Eugene, OR), according to the manufacturer's instructions.

## Results

### Experimental conditions for MALDI MS analysis of low-mass ions in urine

**Figure 1:**
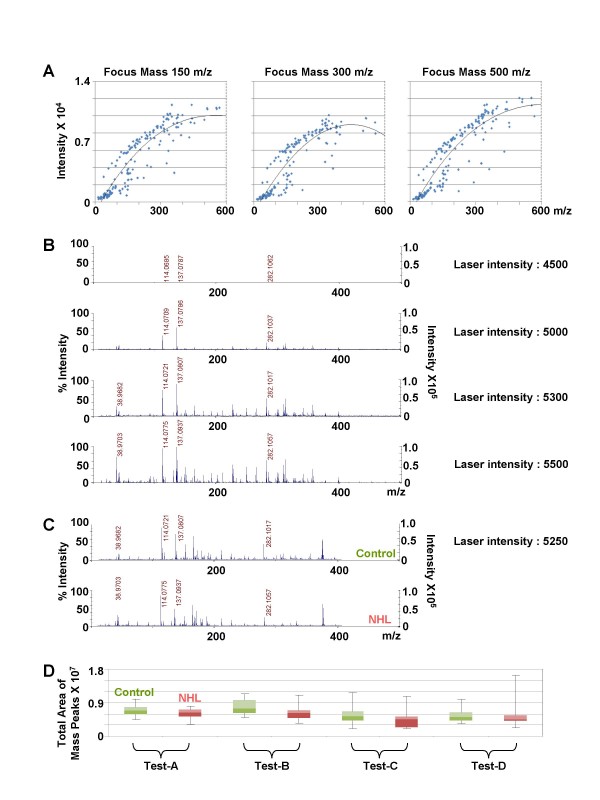
**Selection of two low-mass ions with differential peak areas in urines from controls and NHL patients after establishing MALDI-MS conditions for low-mass-ion profiling**. **A**. The resolution of mass peaks acquired at variable focus mass. Linear resolution of low-mass peaks was obtained at a focus mass of 500 m/z. **B**. The effect of laser intensity on overall mass spectrum acquisition. Laser intensity was positively correlated with the area of low-mass peaks (< 1000 m/z). Because low-mass peaks with the highest intensity become saturated at a laser intensity of 5500, the laser intensity for data acquisition was fixed at 5250. **C**. Typical pattern of mass spectra from urines from controls and NHL patients. **D**. Box plot of total area of low-mass peaks (< 1000 m/z). The total area of low-mass peaks was obtained from four replicate experiments (cases A to D) using urines from 12 controls (green) and 11 NHL patients (pink).

To minimize experimental error, we tested variable parameters, including focus mass, laser intensity, target plate and data-acquisition time. Ideal focus mass and laser intensity for analyzing low-mass ions (< ~1000 m/z) were fixed at 500 m/z and 5250, respectively (Figure 1A, B and 1C). Using these focus mass and laser intensity settings, we analyzed one urine sample four times under different target and data acquisition times. Low-mass peaks that were not significantly affected by experimental conditions were selected and subsequently evaluated for their potential ability to discriminate NHL from normal controls (Figure 1D).

### Selection of low-mass ions differentially present in control and NHL urines

**Figure 2:**
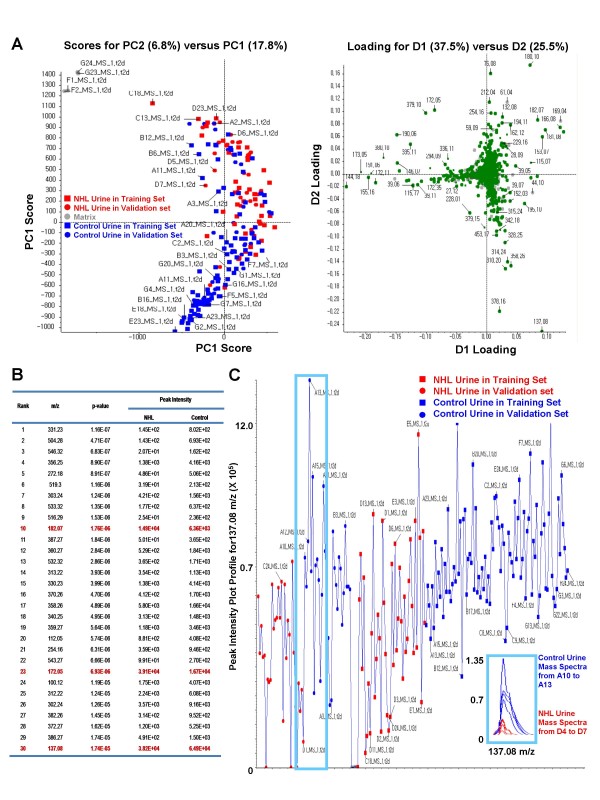
**Discriminating NHL patients from controls based on low-mass ions present in urine**. **A**. Principal components analysis (PCA). The first principle component (PC1) explains the greatest amount of variance, and PC2 represents the next largest amount. *Left panel: *Unsupervised PCA showing that profiling of low-mass ions in urine may discriminate NHL patients. *Right panel*: Supervised PCA describing low-mass ions with differential mass-peak intensities in urines from controls and NHL patients. **B**. Thirty low-mass ions selected by *t*-test analysis. **C**. Profile plot focused on 137.08 m/z peaks. The profile plot demonstrates a higher peak intensity of the 137.08-m/z ion in urines from NHL patients compared to that in controls in both training and validation groups. Lower panel describes overlapped mass spectra of four urine samples from NHL patients and controls from blue box in profile plot.

Using MarkerView™ statistical software, we compared the patterns of low-mass ion spectra in urines of 30 normal and 30 NHL patients. Contamination by the chemical matrix used in MALDI-TOF analysis was ruled out based on a statistical analysis (data not shown). A principal component analysis (PCA) showed differential peak patterns of low-mass ions in control and NHL urines (Figure 2A). Among the low-mass ions ranked in the top 30 by PCA (Figure 2B), three with the highest peak intensity were selected for further analysis. The peak intensity of the 137.08-m/z ion was much lower in urines from NHL patients than in normal urines (P < 0.001) (Figure 2B &2C), whereas those of the other two selected low-mass ions (172.07 and 182.07 m/z) were higher in urines from NHL patients (both P < 0.001) (Figure 2B). A profile plot of 137.08-m/z peaks suggested that this ion has discriminating power for NHL screening (Figure 2C).

### Identifications for the 137.1 m/z ion in urine as hypoxanthine

**Figure 3:**
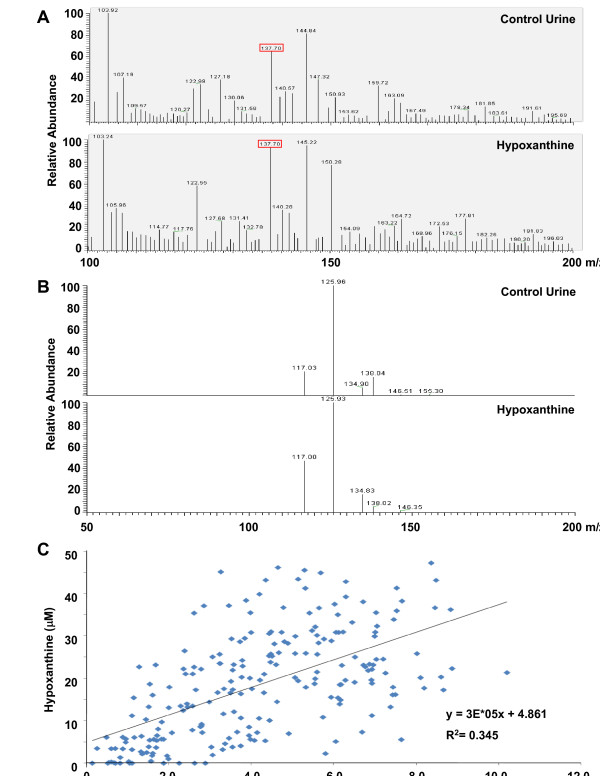
**Identical ESI-MS/MS pattern obtained from 137.08 m/z ion in urine and hypoxanthine**. **A**. Mass shift of the 137.08-m/z ion in urine in LTQ-XL analysis. In a direct urine analysis without LC separation, the urine candidate 137.08-m/z ion and hypoxanthine (HX) were monitored as 137.70 m/z. **B**. The ESI-MS/MS pattern of the 137.08-m/z ion in urine was identical to that of HX. **C**. Mass-peak area of the 137.08-m/z ion and HX concentration were positively correlated; however, this relationship did not reach statistical significance.

Candidate metabolites corresponding to the 137.08-m/z ion were searched using the Human Metabolome Database (HMDB). Many metabolites with a mass tolerance of 137.08 ± 0.05 (137.08 m/z was rounded off to three decimal places) were searched in positive mode. Among these, only eight appeared as an M+H adduct (Table 2). Because it is a representative metabolite in urine metabolic pathways, Hypoxanthine was tested first to determine whether it corresponded to the 137.08 m/z mass peak in urine. To assure the best possible MS/MS analysis of low-mass ions, we employed ESI LC-MS/MS (LTQ-XL, Thermo Fisher Scientific Inc., Waltham, MA), monitoring the mass-shift of hypoxanthine and the 137.08-m/z ion in urine at 137.70 m/z (Figure 3A). Interestingly, the MS/MS pattern of both low-mass ions was identical (Figure 3B). In contrast, the MS/MS patterns of other candidate metabolites, such as N-methylnicotinamide (Table 2), differed from that of the 137.1-m/z ion in urine (data not shown), and were thus ruled out.

**Table 2:** Metabolites with 137.07 ± 0.05 m/z in a positive-mode mass detection

HMDB ID	Common Name	Chemical Formula	Adduct MW (Da) [Matching HMDB MW]	MW Difference (Da) [QueryMass - AdductMass]	Adduct
HMDB03152	N-Methylnicotinamide	C7H8N2O	137.070938 [136.063660]	0.004333	M+H [1+]
HMDB00209	Benzeneacetic acid	C8H8O2	137.059708 [136.052429]	0.006897	M+H [1+]
HMDB01326	Phenyl acetate	C8H8O2	137.059708 [136.052429]	0.006897	M+H [1+]
HMDB11659	2-Methylerythritol	C5H12O4	137.080841 [136.073563]	0.014236	M+H [1+]
HMDB01216	Tetrahydropteridine	C6H8N4	137.082184 [136.074905]	0.015579	M+H [1+]
HMDB00157	Hypoxanthine	C5H4N4O	137.045792 [136.038513]	0.020813	M+H [1+]
HMDB00613	Erythronic acid	C4H8O5	137.044449 [136.037170]	0.022156	M+H [1+]
HMDB00943	Threonic acid	C4H8O5	137.044449 [136.037170]	0.022156	M+H [1+]

M+H[1+] adducts only are listed.

**Figure 4:**
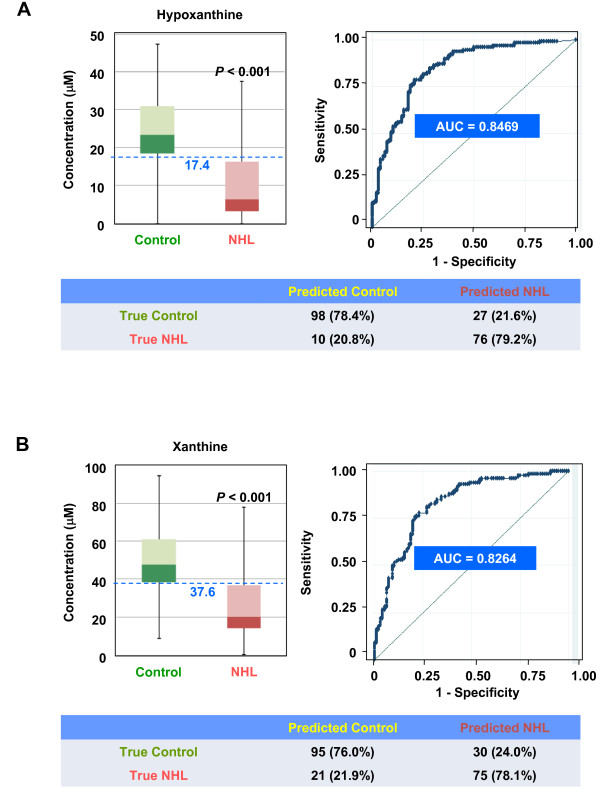
**Significant decrease of hypoxanthine and xanthine in NHL urine**. The levels of hypoxanthine (HX) (**A**) and its oxidative product, xanthine (X) (**B**) were significantly lower in NHL urines (both P < 0.001). Area under the curve, sensitivity and specificity obtained from blind validation of 221 urines samples (125 control, and 96 NHL) are shown.

The mass-peak area of the 137.08-m/z ion and hypoxanthine were correlated with one another (Figure 3C), but this relationship did not reach statistical significance. Mass accuracy (Figure 3A &3B) and the relationship between mass-peak area of the 137.08-m/z ion and hypoxanthine concentration (Figure 3C) suggested that differential levels of HX in control and NHL urines resulted in a change in the mass-peak area of the 137.08-m/z ion. Despite this unsatisfied statistical relationship, hypoxanthine was significantly reduced in NHL urines (P < 0.001); at a 17.4-μM cutoff, specificity and sensitivity were 78.4% and 79.2%, respectively (Figure 4A). Furthermore, xanthine produced from hypoxanthine by xanthine oxidase was also lower in NHL urines, and showed a similar NHL-discriminating power (76.0% specificity and 78.1% sensitivity; Figure 4B).

### Clinicopathological relevance of NHL discriminating low-mass ions in urine

Neither of the two NHL-discriminating low-mass ions, hypoxanthine and xanthine, was significantly correlated with individual histologic types, cancer stage, extra-nodal involvement, Eastern Cooperative Oncology Group score (ECOG performance), international prognostic index (low, low/intermediate vs. high/intermediate, high), bone marrow involvement (absent vs. present), first response to chemotherapy (complete response, partial response vs. stable disease, progressive disease) or survival (data not shown).

## Discussion

Previous efforts to find diagnostic factors for NHL have yielded disappointing results compared with studies reporting prognostic factors with clinicopathological correlations [5,6]. Our previous study showed that the urine level of IL-8 normalized to creatinine could be a possible biomarker with the capacity to discriminate NHL patients from normal controls; thus, we expected that urine might be a valuable biological source for diagnostic markers for NHL [7].

In the present study, we sought to develop a new diagnostic approach for NHL, using MALDI-MS analysis to translate the information of low-mass ions (i.e., < ~1000 m/z) present in urine samples into a tool capable of discriminating NHL patients from normal individuals. There are two motivations for our focus on this low-mass range. The first is the existence of valuable information in the low-mass range, analyzed by mass spectrometry, that has not yet been systematically exploited. Second, ions in the low-mass range provide an enormous amount of information about biological changes that originate from alterations in gene and protein expression. We hypothesized that a new non-invasive cancer-screening protocol could be established if low-mass-ion data were properly collected, statistically translated, and analyzed by MALDI-MS.

Interestingly, a low-mass ion appeared at 137.08 m/z that was significantly different in urine samples from NHL patients and controls (Figure 2B &2C). Searching the Human Metabolome Database (HMDB) yielded a list of candidate metabolites corresponding to the 137.08-m/z ion (Table 2). An ESI-MS/MS analysis of low-mass ions in urine identified 137.08 m/z as hypoxanthine (Figure 3B and 3C). Consistent with this identification, the concentration of hypoxanthine in urine compared favorably with the MALDI-MS profile of the 137.08-m/z ion (Figure 3C).

The Low-mass ion profiling is absolutely depends on the accurate mass measuring technology. Recent mass spectrometers employing either MALDI-, or ESI-based technology provide very accurate mass information. Using this advanced technology, we herein were able to suggest a possible application of low-mass ion profiling for cancer screening. However, a couple of problems are still remained. First, software for low-mass ion profiling is still incomplete. During the normalization process of mass spectra obtained from each individual samples, software sometimes collects the noise on the mass spectrum as a low-intensity peaks with discriminating power. To prevent this nose picking, low-mass ions ranked by software have to be checked again on the raw mass spectra. Furthermore, the low-mass ions with low-intensity, even they have a great discriminating power, may not be proper for identification. This is the reason why the low-mass ion with 137.08 m/z was selected for identification since it showed highest intensity among the low-mass ions ranked within 30^th ^(Figure 2B).

In patients with gastric cancer or colorectal cancer, purine bases in plasma have been reported to increase in association with a decrease in purine base excretion [8]. Whether hypoxanthine and xanthine levels are altered in urine samples from gastrointestinal cancer patients is still a matter of debate [8]. In plasma from children with acute lymphoblastic leukemia or NHL, hypoxanthine levels were reported to be higher than those in healthy adult controls; these elevated plasma hypoxanthine levels decreased after methotrexate infusion [9]. However, a change in urine levels of hypoxanthine has not yet been reported.

At present, we are unable to explain the underlying mechanism for the change in hypoxanthine, but one possibility may be found in alterations of purine metabolism that occur during tumor development. Intracellular concentrations of hypoxanthine and xanthine are inversely related to adenylate energy changes and, therefore, to the energy currency of cellular ATP [10]. Recently, a classical antifolate has been shown to possess cytotoxic activity against human prostatic cancer cell lines that lacked hypoxanthine, whereas growth was maintained in tumors with hypoxanthine [11]. We reasoned that the level of hypoxanthine in NHL urines might decrease due to consumption by tumor cells. However, a recent study has shown that urinary hypoxanthine is significantly increased when tumor development in mesothelioma-transplanted nude mice was maximized [12]. In addition, changes in the activity or expression of enzymes involved in hypoxanthine or xanthine metabolism, which might affect hypoxanthine and xanthine levels in NHL urines, cannot be ruled out. For example, xanthine oxidoreductase, a key enzyme in the degradation of DNA and RNA, is associated with histological grade of differentiation and extent of disease in colorectal cancer [13], as well as the migratory activity of human breast cancer cells [14,15].

## Conclusions

The present study represents a good example of low-mass-ion profiling in the setting of disease-screening using urine samples. This technique can be a powerful, non-invasive diagnostic tool with high sensitivity and specificity for NHL screening. Furthermore, hypoxanthine identified in this study may be a useful single urine marker for NHL screening. However, the biochemical mechanism responsible for the decreased levels of hypoxanthine and xanthine in urine samples from NHL patients remains to be elucidated.

## Competing interests

The authors declare that they have no competing interests.

## Authors' contributions

BCY and HSE conceived of the study, and participated in its design, coordination and data interpretation. SYK, WSP, TY and HSE performed pathological studies. SYK and SP carried out statistical analyses. SGJ, KHK and SAA carried out the mass analyses and metabolite identification. BCY drafted the manuscript. SP, WSP and TY advised on manuscript content and in critical revisions. BCY and HSE jointly wrote the final versions of the manuscript. All authors read and approved the final manuscript.

## Pre-publication history

The pre-publication history for this paper can be accessed here:

http://www.biomedcentral.com/1471-2407/10/55/prepub
